# Michael J. Duff: a personal reminiscence

**DOI:** 10.1098/rspa.2022.0167

**Published:** 2022-03

**Authors:** Steven Weinberg

**Affiliations:** Department of Physics, The University of Texas at Austin, Austin, TX, USA

I first became acquainted with Mike Duff in the late 1970s. I was developing an idea known as ‘asymptotic safety’. According to this idea, all forces including gravitation are described by a quantum field theory with an infinite number of energy-dependent couplings, all couplings that are allowed by symmetry principles. With increasing energy, these couplings tend to run to infinity, except that there may be a finite-dimensional surface of coupling trajectories that are safely attracted to a fixed point as the energy goes to infinity. From the work of Wilson and Fisher, I knew that in scalar field theories in 2+ε dimensions there is such a fixed point. My problem was to find the conditions under which this would still be true in a theory with gravitons and scalar, vector and spinor fields. Eventually, I found the solution: ask Mike Duff!

As I acknowledged in my article on asymptotic safety, Mike’s expertise was tremendously helpful to me. In the following years, I became increasingly aware of his work on supergravity and ‘Kaluza–Klein’ theories in higher dimensional space–times. So I was delighted when in the early 1980s he agreed to visit our Theory Group here in Austin.

His visit was a smashing success. He gave a superb series of lectures on Kaluza-Klein theories. It was also fun to have him in town. I recall that once my wife and I went with Mike and Chris Pope to wander about Sixth Street, Austin's Barbary Coast. Sitting at a table in a honky-tonk we were visited by a young lady, who offered her professional services. Mike was wonderfully suave and polite in declining the offer.

Since then Mike and I have been mostly on other sides of the water, and I have had little chance to spend time with him. I have of course been aware of his brilliant work on superstrings, supermembranes and all that, which I assume will be described by other contributors to this volume. It was a great pleasure to see him again on my rare visits to London, UK, most recently for a meeting at Imperial College. For personal and professional reasons, I count myself fortunate to know him.

## Data Availability

This article has no additional data.

